# 3′,4′-Dihydroxyflavonol Reduces Superoxide and Improves Nitric Oxide Function in Diabetic Rat Mesenteric Arteries

**DOI:** 10.1371/journal.pone.0020813

**Published:** 2011-06-06

**Authors:** Chen-Huei Leo, Joanne L. Hart, Owen L. Woodman

**Affiliations:** School of Medical Sciences, Health Innovation Research Institute, Royal Melbourne Institute of Technology (RMIT) University, Bundoora, Victoria, Australia; University of London, United Kingdom

## Abstract

**Background:**

3',4'-Dihydroxyflavonol (DiOHF) is an effective antioxidant that acutely preserves nitric oxide (NO) activity in the presence of elevated reactive oxygen species (ROS). We hypothesized that DiOHF treatment (7 days, 1 mg/kg per day s.c.) would improve relaxation in mesenteric arteries from diabetic rats where endothelial dysfunction is associated with elevated oxidant stress.

**Methodology/Principal Findings:**

In mesenteric arteries from diabetic rats there was an increase in ROS, measured by L-012 and 2',7'-dichlorodihydrofluorescein diacetate fluorescence. NADPH oxidase-derived superoxide levels, assayed by lucigenin chemiluminescence, were also significantly increased in diabetic mesenteric arteries (diabetes, 4892±946 counts/mg versus normal 2486±344 counts/mg, n = 7–10, p<0.01) associated with an increase in Nox2 expression but DiOHF (2094±300 counts/mg, n = 10, p<0.001) reversed that effect. Acetylcholine (ACh)-induced relaxation of mesenteric arteries was assessed using wire myography (pEC_50_ = 7.94±0.13 n = 12). Diabetes significantly reduced the sensitivity to ACh and treatment with DiOHF prevented endothelial dysfunction (pEC_50_, diabetic 6.86±0.12 versus diabetic+DiOHF, 7.49±0.13, n = 11, p<0.01). The contribution of NO versus endothelium-derived hyperpolarizing factor (EDHF) to ACh-induced relaxation was assessed by evaluating responses in the presence of TRAM-34+apamin+iberiotoxin or N-nitro-L-arginine+ODQ respectively. Diabetes impaired the contribution of both NO (maximum relaxation, R_max_ diabetic 24±7 versus normal, 68±10, n = 9–10, p<0.01) and EDHF (pEC_50_, diabetic 6.63±0.15 versus normal, 7.14±0.12, n = 10–11, p<0.01) to endothelium-dependent relaxation. DiOHF treatment did not significantly affect the EDHF contribution but enhanced NO-mediated relaxation (R_max_ 69±6, n = 11, p<0.01). Western blotting demonstrated that diabetes also decreased expression and increased uncoupling of endothelial NO synthase (eNOS). Treatment of the diabetic rats with DiOHF significantly reduced vascular ROS and restored NO-mediated endothelium-dependent relaxation. Treatment of the diabetic rats with DiOHF also increased eNOS expression, both in total and as a dimer.

**Conclusions/Significance:**

DiOHF improves NO activity in diabetes by reducing Nox2-dependent superoxide production and preventing eNOS uncoupling to improve endothelial function.

## Introduction

Endothelial dysfunction, characterized by the impairment of endothelium-dependent relaxation, is recognised as a critical and initiating factor in the development of diabetes-induced vascular complications [1], [2]. Oral hypoglycaemic agents and insulin have been used to treat diabetes but they do not prevent the development of diabetic vascular complications [3], [4], [5]. In diabetes, hyperglycaemia-induced oxygen radical generation, mainly superoxide anion radicals, play a key role in the pathogenesis of vascular complications [6], [7], [8]. Despite this, clinical trials with antioxidants have failed to clearly demonstrate any beneficial effect on vascular function [9], [10]. More effective antioxidants, acting perhaps by targeting the specific sources of reactive oxygen species (ROS) might prove more beneficial than direct scavenging strategies [8], [11], [12]. Potential targets for pharmacological therapies include NADPH oxidase, endothelial nitric oxide synthase (eNOS) and mitochondria all of which have been reported to be sources of increased ROS in diabetes [2], [13].

Flavonols are one class of a large family of plant-derived polyphenolic compounds known as flavonoids. They exhibit a variety of biological actions such as antithrombotic, anti-inflammatory, antioxidant and vasorelaxant effects [14], [15]. There is growing evidence that consumption of a flavonoid-rich diet reduces the risk of cardiovascular disease states associated with overproduction of ROS [16], [17]. In addition, a number of studies have shown that, in animal models of diabetes, chronic treatment with the flavonol quercetin preserves endothelial function [18], reduces pancreatic beta-cell death [19] and protects against diabetic nephropathy [20].

Structure-activity relationship studies have demonstrated that a synthetic flavonol, 3',4'-dihydroxyflavonol (DiOHF) is significantly more potent than a number of natural flavones and flavonols in its antioxidant ability [21], [22]. In addition, the antioxidant ability of DiOHF has been shown to preserve endothelial function in the aorta in the presence of oxidative stress [23], [24]. Furthermore, DiOHF effectively reduces oxidative stress related impairment of cardiovascular function after ischaemia/reperfusion in rats [23] and sheep [25], [26] and in diabetic aorta [27]. Recently, we have shown the acute antioxidant activity of DiOHF is able to restore endothelial function in the diabetic microvasculature [28] but it is not known whether treatment with DiOHF is able to inhibit the sources of ROS production[29] in the diabetic microvasculature to improve endothelial function.

Diabetes-induced endothelial dysfunction is due to the impairment of both NO-mediated and endothelium-dependent hyperpolarizing factor (EDHF)-type relaxation, which is accompanied by an increase in Nox2-derived superoxide production and eNOS uncoupling in the mesenteric artery [30]. Therefore, the aim of the present study was to investigate whether short term *in vivo* treatment with DiOHF preserves microvascular endothelial function in mesenteric artery from type 1 diabetic rats and, if so, whether it acts via direct scavenging of ROS and/or by inhibiting the sources of ROS production in the diabetic microvasculature. In addition, we also sought to verify whether the antioxidant activity of DiOHF treatment would have any effect on NO-mediated relaxation and/or EDHF-type relaxation in the diabetic arteries.

## Results

### Body weights and blood glucose

The body weight gained, blood glucose and HbA_1c_ levels of the rats are shown in Table 1. 8 weeks after treatment with streptozotocin or vehicle, the body weight gained in normal rats was significantly greater than in diabetic rats (Table 1). The blood glucose and HbA_1c_ level of diabetic rats were significantly greater than normal rats. Treatment with DiOHF had no significant effect on body weight gain, blood glucose or HbA_1c_ levels in normal rats, but significantly increased the body weight gain in diabetic rats. In comparison to DiOHF-treated normal rats, the diabetic rats that were treated with DiOHF had significantly lower body weight gain and significantly greater blood glucose and HbA_1c_ level (Table 1).

**Table 1:** Mean body weight gained, blood glucose and glycated haemoglobin levels at the end of the experiment of normal and diabetic rats with or without 3′, 4′-dihydroxyflavonol (DiOHF, 1 mg/kg s.c. daily for 7 days) treatment.

	8 weeks after vehicle or STZ treatment							
	n	Normal	n	Normal+DiOHF	n	Diabetic	n	Diabetic+DiOHF
**Body weight gained (g)**	22	307±12	19	286±5	19	126±10*	20	177±10† ‡
**Blood glucose (mM)**	22	8.3±0.6	19	7.3±0.6	19	32.4±0.3*	20	30.2±0.9‡
**HbA1_c_ (%)**	8	6.1±0.2	7	5.8±0.2	10	13.5±0.5*	10	12.6±0.6‡

n =  the number of rats. * Significantly different from normal group (Bonferroni's test, p<0.05), ^†^ Significantly different to diabetic group, p<0.05, Bonferroni's test. ^‡^ Significantly different to Normal+DiOHF group, p<0.05, Bonferroni's test. Results are shown as mean±SEM.

### Effect of DiOHF on ROS production

**Figure 1:**
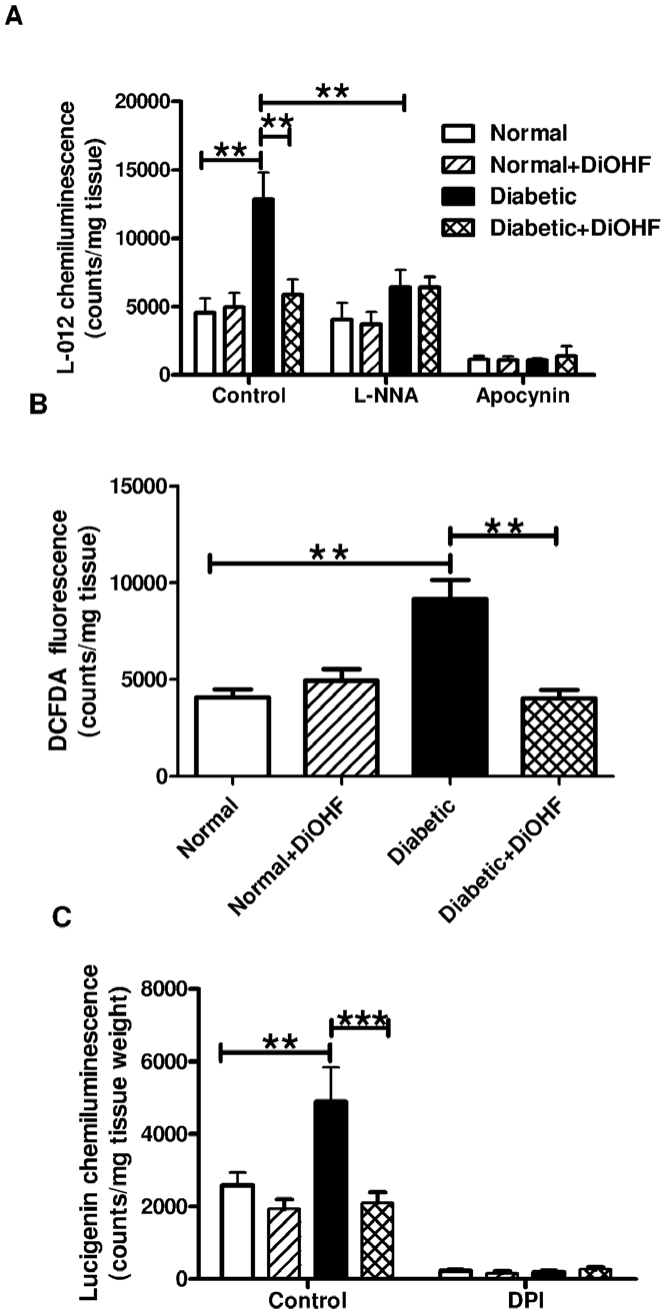
ROS measurement in intact mesenteric arteries. A, Superoxide, B, DCFDA-induced flurorescence levels and C, NADPH-oxidase activity was increased in diabetic arteries which was reduced with DiOHF treatment. A, Superoxide levels in diabetic arteries were attenuated by the presence of L-NNA (100 µM), indicating eNOS uncoupling. n = 9–10 experiments. * p<0.05, ** p<0.01, *** p<0.001.

The ROS level in mesenteric arteries was measured by L-012 chemiluminescence and 2',7'-dichlorodihydrofluorescein diacetate (DCFDA) fluorescence. The superoxide and DCFDA-induced fluorescence levels from diabetic rats were significantly higher than in normal rats (Figure 1). 7 days treatment with DiOHF attenuated the generation of superoxide (Figure 1A) and DCFDA-induced fluorescence levels (Figure 1B) in diabetic rats, but had no effect in normal arteries. The presence of L-NNA attenuated the generation of superoxide in diabetic arteries, but had no effect in either normal or DiOHF-treated arteries. Apocynin, a ROS scavenger, attenuated the production of superoxide in arteries from all groups (Figure 1A).

The level of NADPH oxidase-driven superoxide production detected by L-012-enhanced chemiluminescence in mesenteric arteries from diabetic rats was significantly increased in comparison to normal rats (Figure 1C). DiOHF treatment of the rats did not affect superoxide production by mesenteric arteries from normal rats but significantly reduced levels generated by diabetic mesenteric arteries to levels observed from normal rats. In all groups, NADPH oxidase-driven superoxide production from mesenteric arteries could be inhibited by diphenyl iodonium, a flavoprotein inhibitor that inhibits NADPH oxidase (Figure 1C).

### Effect of DiOHF on vascular function

**Figure 2:**
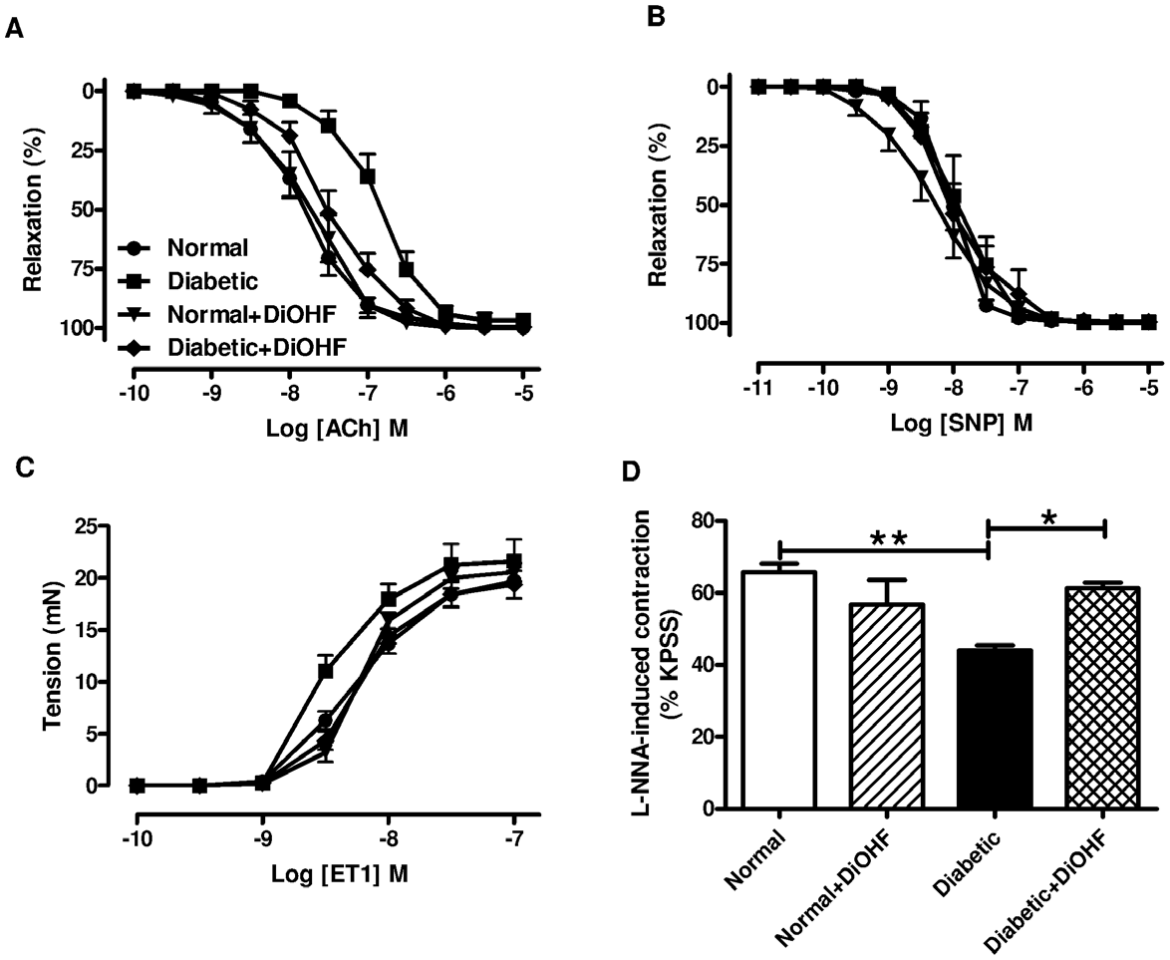
Vascular function in mesenteric arteries. Cumulative concentration-response curves to A, ACh, B, SNP, C, ET1 and D, basal NO release in endothelium-intact mesenteric arteries. In each group of experiments (A, B), mesenteric arteries were precontracted with phenylephrine to similar level: (A) normal 59±3, normal+DiOHF 59±3, diabetic 62±2, diabeticDiOHF 60±3, (B) normal 58±2, normal+DiOHF 57±3, diabetic 61±3, diabeticDiOHF 59±4%KPSS, n = 5–12 experiments. Results are shown as mean±SEM. * p<0.05, ** p<0.01. See Table 2 or results section for pEC_50_ and R_max_ values.

The level of the reference contraction, high K^+^ physiological saline solution (KPSS, 123 mmol/l), was not affected by diabetes or DiOHF treatment (Figure S1). Diabetes significantly reduced the sensitivity, but not the maximum relaxation, to ACh in mesenteric arteries (Figure 2A, Table 2), whereas the sensitivity (diabetic, 7.96±0.20 vs. normal, 7.89±0.16, n = 5–6, p>0.05) and maximum relaxation (diabetic, 100±0% vs. normal, 99±1%, n = 5–6, p>0.05) to sodium nitroprusside (SNP) were not affected (Figure 2B). Treatment of normal rats with DiOHF (1 mg/kg s.c.) for 7 days did not affect responses to ACh, however in mesenteric arteries from diabetic rats treated with DiOHF, the sensitivity to ACh was significantly increased in comparison to the response in mesenteric arteries from untreated diabetic rats (Figure 2, Table 2). DiOHF treatment did not affect responses to SNP in normal rats (pEC_50_ 8.18±0.18, R_max_ 100±0%, n = 9) or diabetic rats (pEC_50_ 7.84±0.18, R_max_ 99±1%, n = 6).

**Table 2:** Effect of L-NNA, ODQ and potassium channel blockers on ACh-induced relaxation of mesenteric arteries from normal and diabetic rats with or without 3′, 4′-dihydroxyflavonol (DiOHF, 1 mg/kg s.c. daily for 7 days) treatment in the presence of indomethacin.

		Normal			Normal + DiOHF			Diabetic			Diabetic + DiOHF	
	n	pEC_50_	R_max_ (%)	n	pEC_50_	R_max_ (%)	n	pEC_50_	R_max_ (%)	n	pEC_50_	R_max_ (%)
Control	12	7.94±0.13	100±0	10	7.78±0.16	100±0	11	6.86±0.12†	97±3	11	7.49±0.13§	100±0
TRAM-34 + apamin	12	7.26±0.19*	91±3	9	6.93±0.22*	92±2	11	6.86±0.16	66±8* †	11	7.04±0.11*	86±3* §
TRAM-34 + apamin+Ibtx	10	6.83±0.15*	68±10*	9	6.84±0.19*	71±11*	10	ND	31±9* †	11	6.98±0.12*	69±6* §
30 mM K^+^	8	6.91±0.10*	78±4*	9	7.28±0.11* †	73±4*	8	6.35±0.07* †	52±5* †	7	6.81±0.13* ‡ §	70±2* §
L-NNA + ODQ	12	7.14±0.12*	100±0	10	7.07±0.15*	98±1	10	6.63±0.15†	97±1	11	6.85±0.12*	99±1
L-NNA + ODQ + TRAM-34 + apamin	10	5.48±0.23*	59±10*	10	6.10±0.30*	70±9*	6	ND	1±1* †	7	ND	2±2* ‡
L-NNA + ODQ + TRAM-34 + apamin+Ibtx	7	ND	3±3*	5	ND	2±2*						

A comparison of the sensitivity (pEC_50_) and maximum relaxation (R_max_) to ACh in the absence (control), or in the presence of TRAM-34 (1 µM)+apamin (1 µM), TRAM-34 (1 µM)+apamin (1 µM) +Ibtx (100 nM), 30 mM K^+^, L-NNA (100 µM)+ODQ (10 µM), L-NNA (100 µM)+ODQ (10 µM)+TRAM-34 (1 µM)+apamin (1 µM) or L-NNA (100 µM)+ODQ (1 µM)+TRAM-34 (1 µM)+apamin (1 µM)+Ibtx (100 nM) in endothelium intact mesenteric arteries. All experiments were conducted in the presence of indomethacin (10 µM). n = the number of experiments.

*Significantly different to control within each group, p<0.05, Dunnet's test,

† Significantly different to normal within inhibitor group, p<0.05, Bonferroni's test.

‡ Significantly different to normal+DiOHF within inhibitor group, p<0.05, Bonferroni's test.

§ Significantly different to diabetic within inhibitor group, p<0.05, Bonferroni's test. Results are shown as mean±SEM, ND =  Not determined.

The diabetic arteries also showed a significant increase in sensitivity to endothelin-1 (ET-1, diabetic, 8.46±0.07 vs. normal, 8.19±0.06, n = 7–8, p<0.05), without affecting the maximum contraction. Treatment with DiOHF had no effect in normal arteries (normal+DiOHF, 8.16±0.03, n = 7), but significantly reduced the sensitivity to ET-1 in diabetic arteries in comparison to untreated diabetic arteries (diabetic, 8.46±0.07 vs. diabetic+DiOHF, 8.20±0.07, n = 7–8, p<0.05), (Figure 2C).

### Relative contribution of NO and EDHF to endothelium-dependent relaxation

In normal mesenteric arteries, ACh-induced relaxation could be partly inhibited by either the combination of a NO synthase inhibitor, N-nitro-L-arginine (L-NNA) and a soluble guanylate cyclase inhibitor, 1H-(1,2,4)-oxadiazolo(4,2-a)quinoxalin-1-one (ODQ), or K_Ca_ channels inhibitors, 1-[(2-chlorophenyl)(diphenyl)methyl]-1H-pyrazole (TRAM-34), apamin and iberiotoxin (Ibtx), to block the intermediate-conductance calcium-activated K^+^ channel (IK_Ca_),small-conductance calcium-activated K^+^ channel (SK_Ca_) and large-conductance calcium-activated K^+^ channel (maxi K_Ca_) respectively, indicating that both NO and EDHF contributed to endothelium-dependent relaxation. However, in diabetic arteries, ACh-induced relaxation could only be inhibited by the K_Ca_ channels inhibitors, suggesting that EDHF was the predominant contributor to endothelium-dependent relaxation in diabetes. In the presence of both NO and EDHF inhibitors, endothelium-dependent relaxation were abolished in all groups of rats (Table 2).

### Effect of DiOHF on NO-mediated relaxation

**Figure 3:**
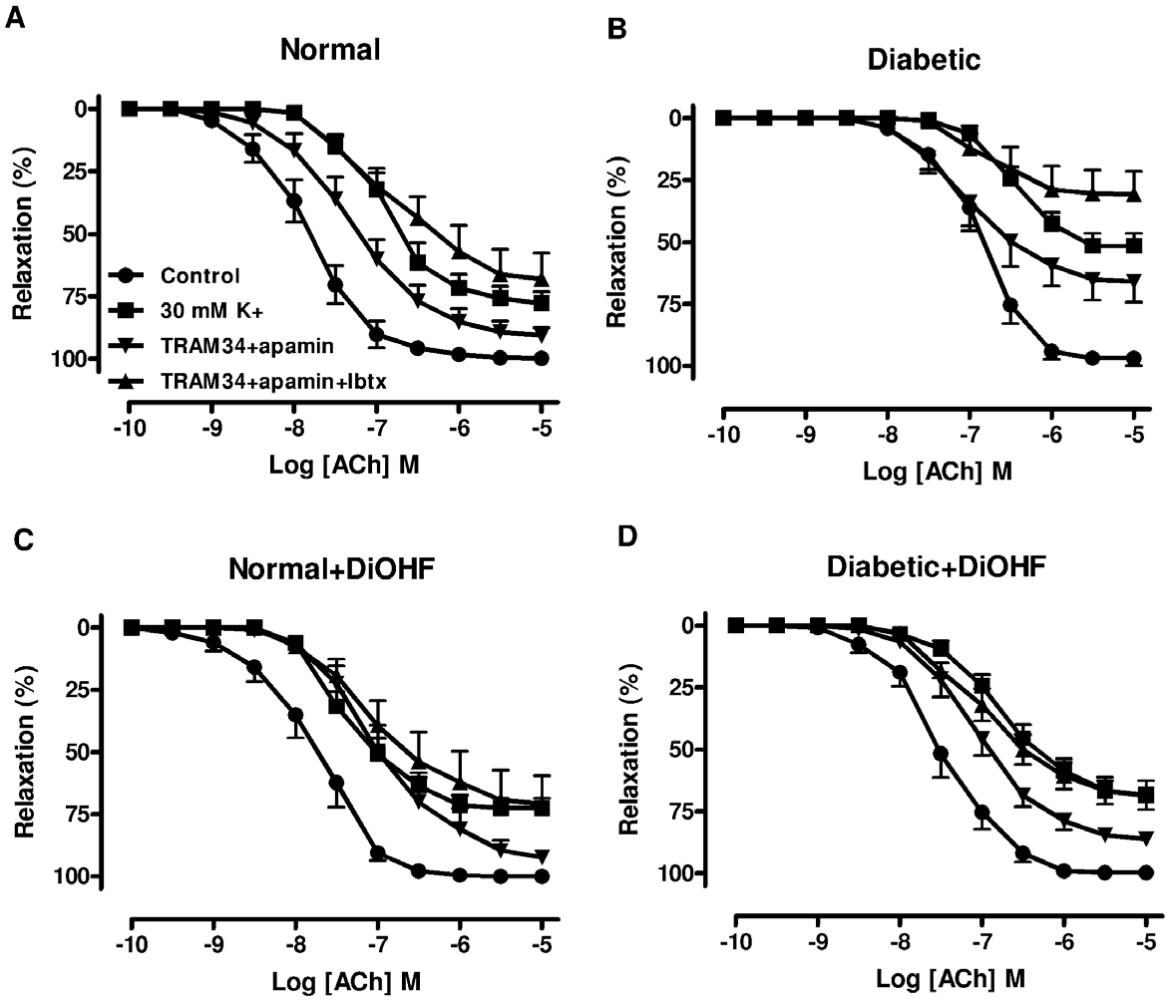
Contribution of NO to endothelium-dependent relaxation in mesenteric arteries. NO-mediated relaxation in mesenteric arteries isolated from A, normal, B, diabetic, C, normal+DiOHF, D, diabetic+DiOHF rats. In each group of experiments, arteries were precontracted to similar level: A, 57±3, B, 58±2, C, 61±4, D, 59±2%KPSS, n = 9–15 experiments. Results are shown as mean±SEM. See Table 2 for values.

To determine the NO-mediated component of the relaxation, responses to ACh were evaluated in the presence of TRAM-34+apamin or TRAM-34+apamin+Ibtx. When these responses were compared between arteries from normal (Figure 3A, Table 2) and diabetic (Figure 3B, Table 2) rats, it is apparent that diabetes decreased the maximum relaxation to ACh in the presence of TRAM-34+apamin (diabetic 66±8% vs. normal, 91±3%, n = 11–12, p<0.05). A similar difference was also apparent in the presence of TRAM-34+apamin+Ibtx (diabetic 31±9% vs. normal, 68±10%, n = 10, p<0.05), indicating that diabetes impaired the contribution of NO to endothelium-dependent relaxation. Treatment with DiOHF (1 mg/kg s.c.) for 7 days had no effect in normal rats but significantly increased the maximum relaxation to ACh (diabetic 66±8% vs. diabetic+DiOHF, 86±3%, n = 11, p<0.05) in the presence of TRAM-34+apamin (Figure 3B, D, Table 2). A similar finding was observed in DiOHF-treated diabetic arteries (Figure 3B, D, Table 2) in the presence of TRAM-34+apamin+Ibtx (diabetic 31±9% vs. diabetic+DiOHF, 69±6%, n = 11, p<0.05), indicating that DiOHF treatment improved the contribution of NO to endothelium-dependent relaxation in diabetic arteries. In addition, in arteries pre-contracted with a depolarizing solution of 30 mM K^+^, to eliminate any contribution of the opening of potassium channels, ACh-induced relaxation was significantly attenuated in diabetic arteries in comparison to normal arteries, but this response was significantly improved by 7 days treatment with DiOHF in diabetic rats (Figure 3, Table 2).

The basal level of NO release was assessed by measuring the contraction induced by L-NNA in arteries with PE-induced tone (i.e. 20% KPSS) (Figure 2D). The L-NNA-induced contraction was significantly greater in arteries from normal rats compared with diabetic rats (normal, 66±2% vs. diabetic, 44±2%, n = 6–7, p<0.05), indicating that diabetes impaired the basal release of NO. Treatment with DiOHF had no effect on L-NNA-induced contraction in normal arteries (normal+DiOHF, 57±7%), but significantly increased the L-NNA-induced contraction in diabetic arteries in comparison to untreated diabetic arteries (diabetic+DiOHF, 61±2% vs. diabetic, 44±2%, n = 5–6, p<0.05).

### Effect of DiOHF on EDHF-type relaxation

To characterise EDHF-type relaxation, the contribution of NO was eliminated by the combination of L-NNA and ODQ. In the presence of L-NNA+ODQ, the sensitivity to ACh was significantly lower in the diabetic compared to the normal arteries (diabetic, 6.63±0.15 vs. normal, 7.14±0.12, n = 10–12, p<0.05), indicating that diabetes impairs the contribution of EDHF to endothelium-dependent relaxation. Treatment of normal and diabetic rats with DiOHF (1 mg/kg s.c.) for 7 days had no significant effect on ACh-mediated EDHF-type relaxation (Table 2).

**Figure 4:**
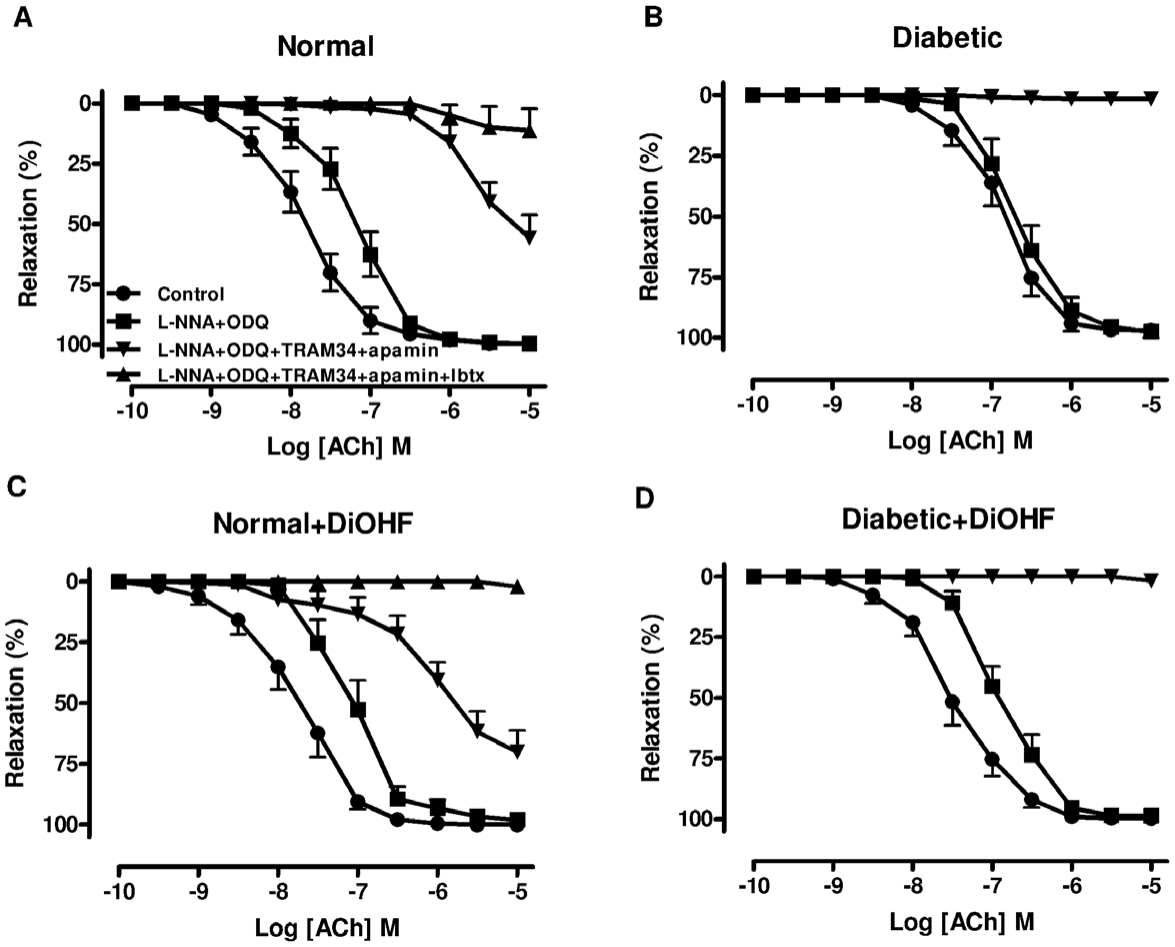
Contribution of EDHF to endothelium-dependent relaxation in mesenteric arteries. EDHF-type relaxation in mesenteric arteries isolated from A, normal, B, diabetic, C, normal+DiOHF, D, diabetic+DiOHF rats. In each group of experiments, arteries were precontracted with phenylephrine to similar level: A, 62±3, B, 63±4, C, 63±4, D, 61±2%KPSS, n = 5–12 experiments. Results are shown as mean±SEM. See Table 2 for values.

The addition of TRAM-34 and apamin to the presence of L-NNA+ODQ, abolished ACh-induced relaxation in either untreated diabetic or DiOHF-treated diabetic arteries. In normal or DiOHF-treated normal arteries, however, ACh continued to cause a maximum relaxation of almost 60% (Figure 4, Table 2). The residual relaxation observed in normal and DiOHF-treated normal arteries could be abolished by the additional presence of Ibtx (Figure 4A, C, Table 2), suggesting an additional role of other endothelium-derived factors to cause relaxation in normal arteries, but not in diabetes.

### Effect of DiOHF on Nox2 and NOS expression, and eNOS uncoupling

**Figure 5:**
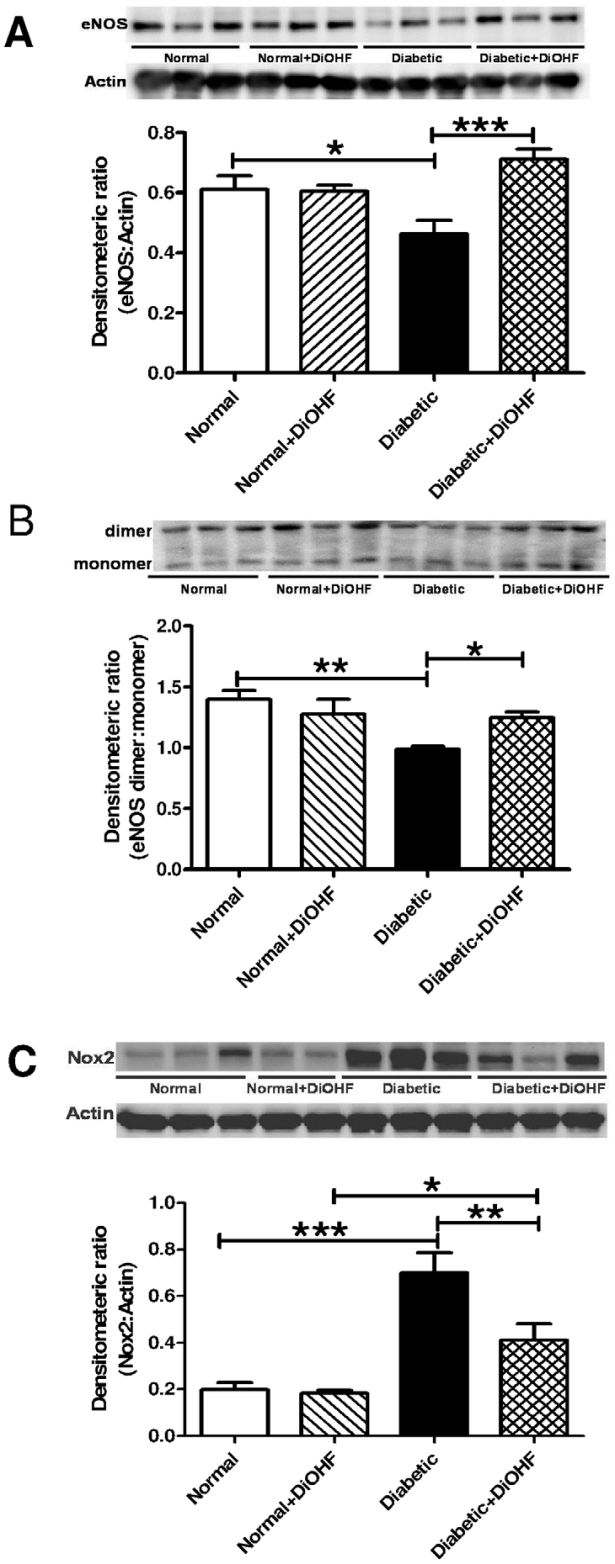
Western blot analysis of protein expression. Western blot of A, eNOS (130 kDa), B, eNOS dimers and monomers (260 kDa) and C, Nox2 (58 kDa) in the normal and diabetic mesenteric arteries with or without DiOHF treatment. Diabetes significantly reduced the expression of eNOS and decreased the proportion of eNOS expressed as the dimer, and increased the expression of Nox2. Treatment with DiOHF significant increased the expression of eNOS, decreased the expression of Nox2 and increased the proportion of eNOS expressed as the dimer. Representative blots were shown on each corresponding graphs. n =  5–6 experiments. Results are shown as mean±SEM. * p<0.05, ** p<0.01, *** p<0.001.

Diabetes significantly decreased the expression of total eNOS in the mesenteric arteries but this was reversed by treatment with DiOHF (Figure 5A). In addition, diabetes significantly decreased the proportion of eNOS expressed as a dimer. Treatment of diabetic rats with DiOHF significantly increased the proportion of eNOS expressed as a dimer (Figure 5B). The expression of Nox2 was also significantly increased in diabetic arteries compared to normal arteries. In DiOHF-treated diabetic arteries, Nox2 expression was significantly reduced, but it remained significantly higher in comparison to DiOHF-normal arteries (Figure 5C). Treatment with DiOHF in normal rats had no effect on eNOS and Nox2 expression and inducible-NOS expression was not detected in any group (data not shown).

## Discussion

This study demonstrates that treatment of type 1 diabetic rats with the synthetic flavonol DiOHF (1mg/kg, per day) for 7 days reduces the levels of vascular oxidative stress and improves endothelium-dependent relaxation in mesenteric arteries. Endothelial dysfunction in the diabetic rats is associated with a decreased contribution of both NO-mediated and EDHF-type relaxation to endothelium-dependent relaxation. Treatment with DiOHF improves NO-mediated relaxation in diabetic rats accompanied by an increased expression of eNOS, reduced eNOS uncoupling and downregulation of Nox2 expression.

### Effect of DiOHF on endothelial function

In the present study, diabetes increases the level of vascular ROS production, associated with an increase in Nox2 expression and a selective impairment of endothelium-dependent relaxation, as we and others have previously described [1], [30], [31], [32]. Treatment with the synthetic flavonol DiOHF (1mg/kg, per day) for 7 days *in vivo* reduces the levels of oxidative stress, at least in part by inhibiting superoxide production by Nox2 and uncoupled eNOS in the diabetic mesenteric arteries, and improves endothelium-dependent relaxation in mesenteric arteries from diabetic rats. These observations are consistent with other studies that have demonstrated that treatment with flavonols in diabetic rats reduces vascular oxidant stress and preserves endothelial function in conduit arteries such as the aorta [18], [27] or renal vasculature [20]. Whilst there are several reports that flavonols attenuate diabetes-induced endothelial dysfunction, the mechanism of the vasoprotective effect of flavonols remain poorly understood, in particular the effect of flavonols on the relative contribution of NO and EDHF to endothelium-dependent relaxation.

### Effects of DiOHF on NO-mediated relaxation

In order to evaluate NO-mediated relaxation, the EDHF-type relaxation was inhibited either by endothelial K_Ca_ channel blockers or by the presence of a depolarizing K^+^ solution. This confirmed an impaired release of NO, as under those conditions the maximum relaxation to ACh was decreased in the diabetic mesenteric arteries in comparison to normal arteries. Treatment with DiOHF *in vivo* for 7 days significantly increased the maximum relaxation to ACh in diabetic arteries in comparison to untreated diabetic rats. In addition to stimulated NO release, basal release of NO was also decreased in diabetic mesenteric arteries as demonstrated by impaired contraction in response to NOS inhibition, and this was also reversed in DiOHF treated diabetic arteries. Hence, treatment with DiOHF preserves NO-mediated relaxation in diabetic small mesenteric arteries.

Consistent with impairment of NO-mediated relaxation, we found that the expression of total eNOS was decreased and uncoupling of eNOS was indicated by the decreased proportion of eNOS expressed as a dimer in the diabetic mesenteric vasculature. This suggests that in the diabetic rats, in addition to producing NO, eNOS is producing superoxide, further demonstrated by a reduction of superoxide levels in the diabetic mesenteric arteries in the presence of NOS inhibition [13], [33]. However, in DiOHF-treated diabetic rats, the expression of eNOS was increased to levels that were comparable to normal rats. In addition, treatment with DiOHF promoted the re-coupling of eNOS, which was shown by the increased proportion of eNOS expressed as a dimer compared to untreated diabetic rats and the lack of inhibitory effect of L-NNA on superoxide production in DiOHF-treated arteries.

A further contributing factor to the restoration of NO activity by DiOHF treatment could be the reduction of vascular superoxide production in diabetic mesenteric arteries, which could then increase the bioavailability of NO by preventing the degradation of NO by superoxide to form peroxynitrite. The reduction of vascular ROS activity in the DiOHF-treated diabetic vasculature could be either due to the rapid free radical scavenging effect of DiOHF to preserve NO bioavailability [23] or by decreasing the expression and/or activity of the enzymatic sources of superoxide production in the vasculature such as the catalytic subunit of NADPH oxidases, Nox2 and its regulatory subunits [34]. Taken together, DiOHF treatment protects the beneficial activity of NO by increasing eNOS expression, preventing eNOS uncoupling and decreasing Nox2-dependent superoxide production in the diabetic mesenteric arteries.

### Effect of DiOHF on EDHF-type relaxation

In the rat mesenteric artery, endothelium-dependent relaxation is mediated by NO, the classical EDHF pathway and there is also a role for the non-classical EDHF pathway [35]. To investigate the role of EDHF, we assessed endothelium-dependent relaxation in the presence of L-NNA and ODQ to inhibit NO synthesis and sGC activity respectively. The guanylate cyclase inhibitor was used in addition to the NOS inhibitor to ensure the inhibition of the actions of NO derived from non-NOS sources such as nitrosothiols, which we have previously reported to act as a NO source in diabetes [33]. In the presence of L-NNA+ODQ, the sensitivity to ACh was decreased in diabetic arteries when compared to normal arteries, indicating that diabetes impaired of the contribution of EDHF-type relaxation, which is consistent with several other studies [36], [37], [38]. Treatment with DiOHF caused a modest improvement in EDHF-type relaxation in diabetic mesenteric arteries, but the change was not statistically significant. It should be noted however that whereas ACh-induced relaxation in the presence of L-NNA+ODQ was impaired in diabetes compared to normal arteries, under the same conditions there was no significant difference in the responses to ACh in arteries from normal and diabetic treated with DiOHF. Thus, the evidence is equivocal as to whether DiOHF improves EDHF-type relaxation in the diabetic mesenteric arteries.

The cause of the impairment of EDHF-type relaxation in diabetes remains controversial [30], [37], [39], [40], [41], [42] but is partly attributed to an overproduction of ROS. Indeed, Ma *et al*., (2008) demonstrated that superoxide anions generated by auto-oxidation of pyrogallol could impair EDHF responses in rat mesenteric arteries, an effect which could be reversed by the acute presence of the flavones, apigenin and/or luteolin, in the tissue bath [43]. In the present study, it is important to note that the final administration of the DiOHF was more than 24 hours before the conduct of all experiments. Although there appears to be no information available regarding the pharmacokinetics of flavonols in rats, in humans it is reported that quercetin reaches peak plasma concentrations within 2–5 hours after oral ingestion and is predominantly excreted in the urine within 5–12 hours [44], [45]. Given the structural similarities between quercetin and DiOHF, it is unlikely that there will be any acute effect of DiOHF during experimentation. Therefore, the *in vivo* effect of DiOHF on EDHF-type relaxation remains to be elucidated.

### Conclusion

We have demonstrated that the synthetic flavonol DiOHF increases NO activity to improve endothelial function in diabetic microvasculature. It was less certain as to whether DiOHF treatment had a beneficial effect on the EDHF-type component of endothelium-dependent relaxation. The protective actions of DiOHF occur through at least two mechanisms. The flavonol is able to rapidly scavenge ROS and/or inhibit the enzymatic source for superoxide production to reduce inactivation of NO. In addition, treatment with DiOHF *in vivo* for 7 days prevents eNOS uncoupling and thus helps to maintain endothelium-dependent relaxation. The beneficial effect of DiOHF to protect NO activity found in this study indicates that it has potential as a therapeutic agent for use in the prevention of the microvascular complications of diabetes.

## Methods

Ethics statement: All procedures were approved by the Animal Experimentation Ethics Committee of RMIT University (approval number 0822) and conformed to the National Health and Medical Research Council of Australia code of practice for the care and use of animals for scientific purposes.

### Induction of diabetes

Male 6–8 week old Wistar rats weighing approximately 200 g (Animal Resource Centre, Perth, WA, Australia) were randomly divided into two groups: normal and diabetic. Type 1 diabetes was induced by a single injection of streptozotocin (STZ, 50 mg/kg) into the tail vein after the rats were fasted overnight. The control groups received an equivalent volume of the vehicle (0.1 mol/l citrate buffer, pH 4.5) alone. Once the rats were rendered diabetic (blood glucose >25 mmol/l) the following week after STZ injection, all diabetic rats were maintained on a low insulin dose (4–5 IU, s.c., 3 injections per week, Protaphane, Novo Nordisk, NSW, Australia) to promote weight gain and reduce mortality [46]. At the end of the experimental period, blood samples were obtained from the left ventricle and the glucose concentration and glycated haemogloblin (HbA_1c_) were measured using a one touch glucometer (Roche, Sydney, NSW, Australia) and Micromat HbA_1c_ analyser (Biorad, Sydney, NSW, Australia) respectively.

### DiOHF treatment

After seven weeks of STZ-induced diabetes, the 2 groups of rats were further divided into 2 groups (Normal, Normal+DiOHF, Diabetic, Diabetic+DiOHF) receiving either vehicle (10% DMSO+90% peanut oil) or (DiOHF, 1mg/kg s.c. per day) for a period of 7 days. The last dose of DiOHF was administered at least 24 hours prior to the start of experimentation.

### Isolation of mesenteric arteries

Eight weeks after STZ treatment, the rats were killed with pentobarbitone sodium (325 mg/kg, i.p, Virbac, Australia). The mesenteric arcade was isolated and immediately placed in ice cold Krebs bicarbonate solution (118 mmol/l NaCl, 4.7 mmol/l KCl, 1.18 mmol/l MgSO_4_, 1.2 mmol/l KH_2_PO_4_, 25 mmol/l NaHCO_3_, 11.1 mmol/l D-glucose, and 1.6 mmol/l CaCl_2_) containing indomethacin (10 µmol/l), a non-selective cyclo-oxygenase (COX) inhibitor, to inhibit the synthesis of prostanoids. Our preliminary data suggested that there is no significant contribution of prostanoids to endothelium-dependent in mesenteric arteries from all groups of rats (data not shown). Small mesenteric arteries (third-order branch of the superior mesenteric artery, internal diameter ∼300 µm) were isolated, cleared of fat and connective tissue, cut into 2 mm long rings and mounted on a Mulvany-Halpern myograph (model 610M, Danish Myo Technology, Aarhus, Denmark). After the arteries were mounted, the vessels were allowed to stabilize at zero tension for 15 min before normalisation. The passive tension-internal circumference was determined by stretching to achieve an internal circumference equivalent to 90% of that of the blood vessel under a transmural pressure of 100 mmHg [47], [48]. All experiments were performed at 37°C and the baths were bubbled with carbogen (95% O_2_ and 5% CO_2_).

### Assessment of vascular reactivity

Thirty minutes after normalization, vessels were maximally contracted with KPSS (123 mmol/l). After several washouts using normal Krebs solution, basal tension was regained. To assess the integrity of the endothelium, mesenteric arteries were precontracted to ∼50–60% of the KPSS response with phenylephrine (0.1–3 µmol/l) and a high concentration of acetylcholine (ACh, 10 µmol/l) was used to relax the artery rings. ACh-induced relaxation was greater than 80% of the precontracted tone in all cases, indicating that the endothelium was functionally intact. After further washouts, arteries were again precontracted to a similar level using phenylephrine (0.1–3 µmol/l) or in some cases 30 mmol/l K^+^, and cumulative concentration-response curves to ACh (0.1 nmol/l–10 µmol/l) and SNP (0.01 nmol/l–10 µmol/l) were determined. In addition, responses to ACh and SNP were examined after 20 minutes incubation with different combinations of L-NNA (100 µmol/l), a non-selective nitric oxide synthase (NOS) inhibitor, ODQ (10 µmol/l), a soluble guanylate cyclase (sGC) inhibitor, TRAM-34 (1 µmol/l), a selective blocker of the intermediate-conductance calcium-activated K^+^ channel (IK_Ca_ or K_Ca_3.1), ibtx (100 nmol/l), a selective blocker of maxi K_Ca_ and apamin (1 µmol/l), a SK_Ca_ inhibitor.

To evaluate the constrictor reactivity, cumulative concentration-response curves to ET-1 (0.1 nmol/l–0.1 µmol/l) were constructed in the absence of indomethacin.

### Assessment of basal release of NO in mesenteric arteries

In another separate set of experiments, the effect of diabetes on basal levels of NO release was also examined in the absence of indomethacin through the addition of L-NNA (100 µmol/l) in endothelium-intact rings precontracted with phenylephrine (10–100 nmol/l) to approximately ∼20% KPSS. Under those conditions a contractile response to L-NNA was considered to reflect the level of the basal release of NO [30].

### Western Blot

Western blots were performed as described previously [30] with the following modifications. Endothelium-intact mesenteric arteries from 2 animals from the same treatment group were pooled and considered as n = 1. Equal amounts of protein homogenate were subjected to SDS-PAGE and western blot analysis with mouse/rabbit primary antibodies (all 1∶1000, overnight, 4°C) against endothelial NO synthase (eNOS), inducible-NOS, Nox2 (all BD Transduction Laboratories, Lexington, KY, USA). To normalize for the amount of protein, membranes were reprobed with a loading control antibody (actin). All proteins were detected by enhanced chemiluminescence (Amersham, GE Healthcare, Sydney, NSW, Australia) after incubation with anti-mouse/rabbit secondary antibody (Millipore, Billerica, MA, USA) for 1 hour at room temperature (1∶2000). All protein bands were quantified by densitometry (Biorad Chemidoc, Sydney, NSW, Australia) and expressed as a ratio of the loading control. To investigate eNOS homodimer formation in the tissue, a non-boiled sample was resolved by 6% SDS-PAGE at 4°C [49], and the membranes were probed and visualized as described above.

### Measurement of ROS in mesenteric artery

Two different methods of ROS measurement were employed. Superoxide production in the mesenteric artery was measured using L-012 as previously described [30]. Mesenteric arteries were incubated at 37°C for 30 minutes in Krebs-HEPES buffer either alone, in the presence of apocynin, a ROS scavenger [50] (300 µmol/l) or L-NNA (100 µmol/l), which determines NOS-derived superoxide production. 300 µL of Krebs-HEPES buffer, containing L-012 (100 µmol/l, Wako Pure Chemicals, Osaka, Japan) and the appropriate treatments were placed into a 96-well Optiplate, which was loaded into a Polarstar Optima photon counter (BMG Labtech, Melbourne, VIC, Australia) to measure background photon emission at 37°C. After background counting was completed, a single ring segment of mesenteric artery was added to each well and photon emission was re-counted. Reactive oxygen species (H_2_O_2_) were measured with DCFDA [51] with the following modification. Rings of mesenteric artery were loaded with DCFDA solution (10 µmol/l) for 60 min, followed by 3 washes in Krebs-HEPES buffer. Background fluorescence was measured after excitation at 485 nm and emission at 520 nm. After being rinsed three times with Krebs buffer to remove excess probe, a single segment of the mesenteric artery was added to each well, and fluorescence intensity was recounted. The luminescence and fluorescence counts were normalized with dry tissue weight.

### NADPH oxidase activity

NADPH oxidase-driven superoxide production in the mesenteric artery was measured using lucigenin-enhanced chemiluminescence. Mesenteric arteries were preincubated for 45 min at 37°C in Krebs–HEPES buffer containing diethylthiocarbamic acid (1 mmol/l), to inactivate superoxide dismutase, and NADPH (100 µmol/l) as a substrate for NADPH oxidase, and either alone or in the presence of diphenylene iodonium (5 µmol/l), as a flavoprotein inhibitor that inhibits NADPH oxidase. 300 µL of Krebs-HEPES buffer containing lucigenin (5 µmol/l) and the appropriate treatments were placed into a 96-well Optiplate, and superoxide production was measured and quantified as previously described [27].

### Reagents

All drugs were purchased from Sigma-Aldrich (St Louis, MO, USA), except for acetylcholine perchlorate (BDH Chemicals, Poole, Dorset, UK), DiOHF (Indofine Chemicals, Hillsborough, NJ, USA) and ODQ (Cayman Chemical, Ann Arbor, MI, USA). All drugs were all dissolved in distilled water, with the exception of indomethacin, which was dissolved in 0.1 mol/l sodium carbonate, L-NNA, which was dissolved in 0.1 mol/l sodium bicarbonate, ODQ and TRAM-34, which were dissolved in dimethyl sulfoxide (DMSO).

### Statistical analyses

All results are expressed as the mean±s.e.m., n represents the number of animals per group or the number of assays when tissue from animals was pooled. Concentration-response curves from rat isolated mesenteric arteries were computer fitted to a sigmoidal curve using nonlinear regression (Prism version 5.0, GraphPad Software, San Diego, CA, USA) to calculate the sensitivity of each agonist (pEC_50_). Maximum relaxation (R_max_) to ACh or SNP was measured as a percentage of precontraction to phenylephrine. Group pEC_50_ and R_max_ values were compared by one-way ANOVA with post-hoc analysis using Dunnett's test or Bonferroni's selected comparison test (normal vs. normal+DiOHF, normal vs. diabetic, normal+DiOHF vs. diabetic+DiOHF and diabetic vs. diabetic+DiOHF) as appropriate. P<0.05 was considered statistically significant.

## Supporting Information

Figure S1 **KPSS induced maximum contraction in mesenteric arteries.** Exposure of mesenteric arteries from normal and diabetic rats with or without 3′, 4′-dihydroxyflavonol (DiOHF, 1 mg/kg s.c. daily for 7 days) treatment to high K^+^ physiological saline solution (KPSS, 123 mmol/l). The contraction to KPSS was not affected by diabetes or DiOHF treatment. Results are shown as mean±SEM. NS =  not significant.
(TIF)
