# Supplementary figures and images for: 3′,4′-Dihydroxyflavonol Reduces Superoxide and Improves Nitric Oxide Function in Diabetic Rat Mesenteric Arteries
Source: PLoS One. 2011 Jun 6;6(6):e20813. doi: 10.1371/journal.pone.0020813 (PMC3108977; doi:10.1371/journal.pone.0020813)

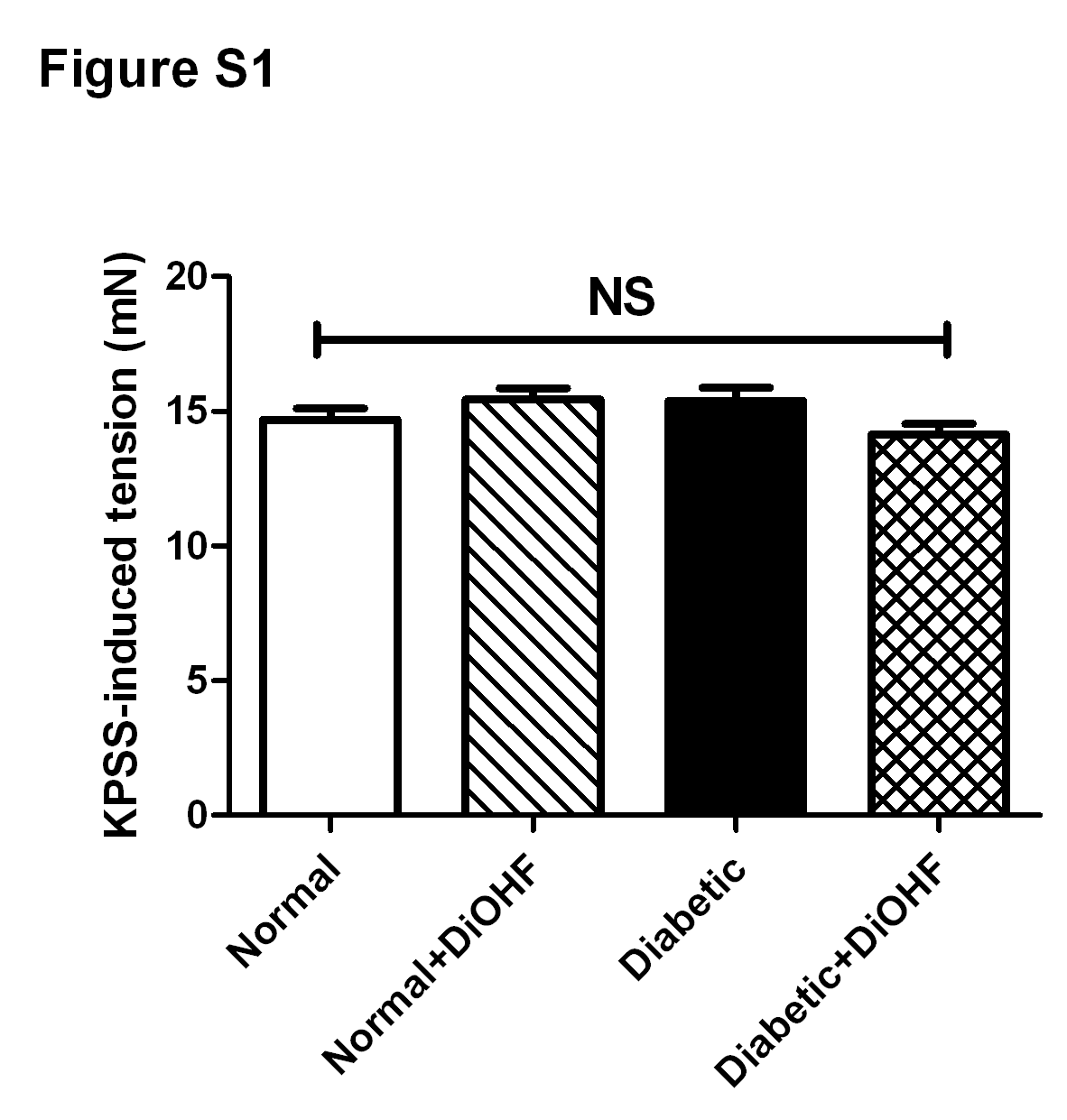

Supplement: Figure S1 — KPSS induced maximum contraction in mesenteric arteries. Exposure of mesenteric arteries from normal and diabetic rats with or without 3′, 4′-dihydroxyflavonol (DiOHF, 1 mg/kg s.c. daily for 7 days) treatment to high K+ physiological saline solution (KPSS, 123 mmol/l). The contraction to KPSS was not affected by diabetes or DiOHF treatment. Results are shown as mean±SEM. NS = not significant. (TIF) [file pone.0020813.s001.tif]
